# Protein arginine methyltransferase expression and activity during myogenesis

**DOI:** 10.1042/BSR20171533

**Published:** 2018-01-10

**Authors:** Nicole Y. Shen, Sean Y. Ng, Stephen L. Toepp, Vladimir Ljubicic

**Affiliations:** Department of Kinesiology, McMaster University, Hamilton, Ontario, Canada

**Keywords:** cell differentiation, Histone, Methylarginine, Mitochondria, PGC-1a, Skeletal muscle

## Abstract

Despite the emerging importance of protein arginine methyltransferases (PRMTs) in regulating skeletal muscle plasticity, PRMT biology during muscle development is complex and not completely understood. Therefore, our purpose was to investigate PRMT1, -4, and -5 expression and function in skeletal muscle cells during the phenotypic remodeling elicited by myogenesis. C_2_C_12_ muscle cell maturation, assessed during the myoblast (MB) stage, and during days 1, 3, 5, and 7 of differentiation, was employed as an *in vitro* model of myogenesis. We observed PRMT-specific patterns of expression and activity during myogenesis. PRMT4 and -5 gene expression was unchanged, while *PRMT1* mRNA and protein content were significantly induced. Cellular monomethylarginines (MMAs) and symmetric dimethylarginines (SDMAs), indicative of global and type II PRMT activities, respectively, remained steady during development, while type I PRMT activity indicator asymmetric dimethylarginines (ADMAs) increased through myogenesis. Histone 4 arginine 3 (H4R3) and H3R17 contents were elevated coincident with the myonuclear accumulation of PRMT1 and -4. Collectively, this suggests that PRMTs are methyl donors throughout myogenesis and demonstrate specificity for their protein targets. Cells were then treated with TC-E 5003 (TC-E), a selective inhibitor of PRMT1 in order to specifically examine the enzymes role during myogenic differentiation. TC-E treated cells exhibited decrements in muscle differentiation, which were consistent with attenuated mitochondrial biogenesis and respiratory function. In summary, the present study increases our understanding of PRMT1, -4, and -5 biology during the plasticity of skeletal muscle development. Our results provide evidence for a role of PRMT1, via a mitochondrially mediated mechanism, in driving the muscle differentiation program.

## Introduction

Protein arginine methyltransferases (PRMTs) are key regulators of important cellular events such as signal transduction, as well as transcriptional activation and repression [1–3]. These enzymes methylate arginine residues by transferring methyl groups from S-adenosyl-l-methionine to terminal guanidino nitrogen atoms of targetted proteins [4]. As a result of PRMT activity, three different methylarginine species are generated, including monomethylarginine (MMA), symmetric dimethylarginine (SDMA), and asymmetric dimethylarginine (ADMA) marks on target proteins [5]. It has recently been demonstrated that the occurrence of arginine methylation is comparable with phosphorylation and ubiquitylation [6], demonstrating the importance of this relatively less understood modification. PRMTs are classified in two groups based on their methylated arginine products: type I PRMTs (i.e. PRMT1, -2, -3, -4, -6, and -8) produce MMA and ADMA, while type II PRMTs (i.e. PRMT5, -7, and -9) generate MMA and SDMA. The importance of PRMT1, -4, and -5 has been demonstrated through mice knockout models where ablation of these enzymes is incompatible with life [7–9]. Furthermore, dysfunction of these PRMTs has been implicated in the most prevalent diseases of Western society, namely cardiovascular disease, diabetes, and cancer [1,10]. For example, overexpression of PRMT1 is linked to lung cancer, while aberrant expression levels of PRMT1, -4, and -5 are observed in breast tumors [5]. Therefore, expanding our understanding of these enzymes will likely have critically important health implications.

Though the presence of arginine methylation in skeletal muscle was first reported almost five decades ago [11], only recently have studies emerged implicating roles for PRMTs in muscle. A number of mechanistic papers have provided a basis for knowledge of PRMT biology in skeletal muscle [3]. For example, PRMT expression, activity, and subcellular localization are dynamic and contribute to the early processes that drive both disuse- and exercise-induced skeletal muscle plasticity *in vivo* [12,13]. Furthermore, the expression and activities of PRMTs are altered throughout myogenesis [14–17]. However, within the context of the developing myogenic program, our understanding of when these adaptations occur, and to what extent, requires further clarification. Reports of PRMT1, -4, -5, and -7 levels during skeletal muscle development have varied from no detected expression [4], to constitutively expressed [4,18,19], to unchanged [17], to increased [16]. Along these lines, the activities of these enzymes throughout myogenesis have also differed between studies. For example, while work from Anthony Imbalzano’s laboratory demonstrated that PRMT4 was only required for late myogenic gene expression [20], recent data from Kawabe et al. [14] showed that PRMT4 methylation regulated Pax7 transcriptional activity, which was necessary for the induction of the early gene *Myf5* (myogenic factor 5). Furthermore, reconciling conflicting reports of PRMT5 function during distinct phases of the myogenic program presents additional challenges to achieve clarity with respect to PRMT activity during skeletal muscle development [4,15,20–22]. Altogether, critical aspects of PRMT biology during myogenesis, such as gene expression and function, are still undefined.

On balance, the conflicting literature on PRMT expression and activity during myogenesis may be due to differences in the PRMTs examined, experimental timecourse utilized, as well as models of muscle differentiation employed in these studies. These inconsistencies make it difficult to elucidate PRMT biology in skeletal muscle. A comprehensive examination of PRMT1, -4, and -5 expression and function throughout a complete timecourse of myogenesis is required in order to clarify and expand our knowledge of the roles of PRMTs in skeletal muscle plasticity. Thus, the purpose of the present study was to examine PRMT1, -4, and -5 expression and function during the conditions of skeletal muscle remodeling evoked during myogenesis. We hypothesized that PRMT expression and activity would be dynamic during muscle development, and that these alterations would exhibit enzyme-specific patterns. A secondary objective, designed to complement a study identifying a critical role for PRMT1 in muscle regeneration that was published during the preparation of this manuscript [17], was to test the requirement of PRMT1 for the progression of skeletal muscle differentiation. We anticipated that the function of PRMT1 during muscle maturation would indeed be essential for optimal muscle development to occur.

## Methods

### C_2_C_12_ muscle cell culture

Commercially available C_2_C_12_ mouse myoblasts (MBs) (American Type Culture Collection, Manassas, U.S.A.) were used in the present study. C_2_C_12_ cells were grown in the presence of growth medium (GM), which comprised Dulbecco’s modified Eagle’s medium (DMEM; Invitrogen Life Technologies, Burlington, Canada) supplemented with 10% FBS (Invitrogen Life Technologies), and 1% penicillin/streptomycin (Invitrogen Life Technologies). The cultured dishes were maintained in a 5% CO_2_ atmosphere at 37°C. Myogenic differentiation was induced when ~90% cell confluence was attained, after which GM was replaced with differentiation medium (DM; DMEM supplemented with 2% horse serum and 1% penicillin/streptomycin; Invitrogen Life Technologies). Cells were grown in DM for 7 days, with DM changed every 48 h. Five experimental timepoints were employed to characterize myogenesis *in vitro*. The first was the MB stage, which was when cells reached ~90% confluence. The second to fifth timepoints occurred throughout the fusion and growth of myotubes (MT), including day 1 (D1) MT (24 h after the transition from GM to DM), day 3 (D3; 72 h after the GM to DM switch), day 5 (D5; 120 h), and day 7 (D7; 168 h). At the specified timepoints, C_2_C_12_ cells were washed three times with Dulbecco’s PBS (HyClone, South Lake, Utah) and processed for subsequent analyses.

### Drug treatments

C_2_C_12_ MBs were cultured until ~90% confluence and then induced to differentiate in medium treated with vehicle (VEH; DM supplemented with DMSO; Invitrogen Life Technologies) or treated with TC-E 5003 (TC-E; DM supplemented with (volume; final concentration) 2.1 µl; 0.1 µM TC-E; Tocris, Bristol, United Kingdom), a highly selective and specific antagonist of PRMT1 [23]. Four experimental timepoints were employed to examine skeletal muscle differentiation, including D1, D3, D5, and D7, after which cells were washed three times with PBS and prepared for analyses.

### Whole cell protein extraction

Cells were scraped in ice-cold RIPA buffer (Sigma–Aldrich, Oakville, Canada) supplemented with cOmplete Mini Protease Inhibitor Cocktail (Roche, Laval, Canada) and PhosSTOP (Roche). Samples were sonicated (Fisher Scientific) 5× 3 s on ice at 100% power. The samples were spun at 20000×***g*** for 15 min. The protein concentrations of the supernatants were determined using the Pierce BCA Protein Assay kit (Thermo Scientific, Rockford, U.S.A.) using BSA as the standard.

### Cell fractionation

Nuclear fractions were isolated from C_2_C_12_ cells at the MB stage as well as at D1, D3, D5, and D7 of muscle differentiation according to procedures as described previously [24], with modifications. Briefly, 500 µl of STM buffer was added to each sample tube and the solution was then homogenized on ice using sonication at 100% power for 10× 2 s, with 30 s between each bout. An additional 200 µl of STM buffer was added before the samples were spun. After centrifuging at 800×***g*** for 15 min, the supernatant and pellet (P0) were separated. The pellet (P0) was resuspended in 1000 µl of STM buffer, vortexed for 15 s, and then centrifuged at 500×***g*** for 15 min. The nuclear pellet (P1) was then suspended in 250–400 µl of STM buffer and spun at 100×***g*** for an additional 15 min while the supernatant was discarded. The sample was again separated and the pellet (P5) was kept; 400 µl of NET buffer was added to the tube before vortexing and then placed on ice for 30 min. The sample was then sonicated at 100% power for 10× 2 s with 30 s in between the bouts. The sample was spun at 9000×***g*** for 30 min before being separated and the supernatant was kept and labeled as the nuclear fraction. Protein concentration of the cellular fractions were determined using the BCA Protein Assay kit.

### Immunoblotting

For whole cell lysates, 20–50 μg of cellular protein was loaded into each lane of 10 or 12.5% polyacrylamide gels and resolved by SDS/PAGE. For nuclear fractions, 40 μg of protein was loaded into each lane. Gels were transferred on to nitrocellulose membranes (Bio–Rad, Mississauga, Canada) after which membranes were stained with either Ponceau S or amido black (Sigma–Aldrich, Oakville, Canada) in order to normalize loading across samples, as previously described [25,26]. Membranes were washed with TBS-Tween 20 ((TBST) (25 mM Tris/HCl (pH 7.5), 1 mM NaCl, and 0.1% Tween 20)) and blocked with 5% skim milk in TBST solution for 1 h. Membranes were subsequently incubated in 5% milk-TBST with primary antibodies overnight at 4°C on a compact digital rocker (Thermo Scientific). The antibodies used were: myogenin (M3559; Dako, Santa Clara, U.S.A.), PRMT1 (P1620; Sigma–Aldrich), which likely recognizes PRMT1v1, PRMT4 (A300-421A; Bethyl, Montgomery, TX), PRMT5 (07-405; EMD Millipore, Massachusetts, U.S.A.), histone 2B (H2B; 8135, Cell Signaling Technology, Massachusetts, U.S.A.), glyceraldehyde 3-phosphate dehydrogenase (GAPDH; ab9483; Abcam, Toronto, Canada), histone 4 arginine 3 asymmetric dimethylation (H4R3^me2a^; 39705, Active Motif, Carlsbad, U.S.A.), histone 3 arginine 8 symmetric dimethylation (H3R8^me2s^) (ab130740, Abcam), histone 3 arginine 17 asymmetric dimethylation (H3R17^me2a^) (ab8284, Abcam), H4 (ab10158, Abcam), H3 (ab1791, Abcam), MMA (mme-R; 8015, Cell Signaling Technology), ADMA (adme-R; 13522, Cell Signaling Technology), SDMA (sdme-RG; 13222, Cell Signaling Technology), peroxisome proliferator-activated receptor γ co-activator-1α (PGC-1α) (AB3242; EMD Millipore), and total oxidative phosphorylation (OXPHOS) cocktail (ab110413; Abcam). After incubation, the blots were washed 3× 5 min in TBST, and appropriate horseradish peroxidise-linked secondary antibodies were applied. The bound antibodies were visualized by ECL (Bio–Rad, Mississauga, Canada) and the membrane was imaged with Alpha Innotech imaging equipment (Alpha Innotech, San Leandro, U.S.A.). ImageJ (NIH) was employed for densitometry.

### Immunofluorescence imaging

Cells were cultured on three 15-mm glass coverslips that were inserted at the bottom of individual 35 × 10 mm^2^ dishes. At each experimental time point, cells were washed three times with PBS and fixed with 4% paraformaldehyde for 30 min. After fixation, cells were washed again in PBS and incubated in 0.25% Triton-X in PBS for 5 min. Following the incubation, blocking was performed with 10% goat serum for 60 min. Subsequently, cells were incubated with embryonic myosin heavy chain (eMHC) primary antibody (Developmental Studies Hybridoma Bank, Iowa, U.S.A.) overnight at 4°C. The next morning, cells were washed in PBS and incubated in secondary antibody (Alexa Fluor goat anti-mouse 594, 1:500 dilution prepared in 1% BSA) for 60 min. Following incubation, the cells were washed in PBS and DAPI (1:20000) was applied and incubated for 5 min. Cells were then washed and the coverslips were carefully removed from the plate. Coverslips were mounted using DAKO fluorescence mounting medium (Agilent Technologies, U.S.A.). Three images were taken from each coverslip. The images were viewed using a fluorescence microscope (Nikon Instruments Canada, Mississauga, ON) at 20× magnification. MT length and width were determined by calculating the average of the five longest and widest MTs using 4×4 eMHC stained immunofluorescence images. The fusion index was calculated by identifying the percentage of eMHC positive cells that had two or more myonuclei. The area fraction was calculated by determining the percentage of eMHC positive cells relative to the total surface area of the image. Cell metrics were determined using Nikon Eclipse Ti-E software (ver. 4.4.2).

### RNA isolation and reverse transcription real-time PCR

Total RNA was extracted from C_2_C_12_ cells using TRIzol reagent (Invitrogen Life Technologies) according to the manufacturer’s protocol. After treating with Turbo DNaseI (Invitrogen Life Technologies) for 30 min, RNA was further purified with RNeasy columns (Qiagen, Toronto, Canada). RNA concentration and integrity (i.e. A260/A230) was measured using a Nanodrop instrument (Thermo Scientific) before being reverse transcribed into cDNA. Superscript III (Invitrogen Life Technologies) was used to synthesize cDNA from total RNA, according to the manufacturer’s instructions. Target cDNA levels were compared by qPCR in reactions containing either SYBR green (Roche Diagnostics, Meylan, France) or GoTaq qPCR Mastermix (Promega, Wisconsin, U.S.A.), forward (F) and reverse (R) primers, and cDNA. qPCR was conducted over 45 cycles at 95°C for 15 s and at 60°C for 1 min, preceded by an initial 95°C for 10 min. The ΔΔ*C*_T_ method was used to calculate the expression of the genes of interest with the average of 18S rRNA (18S), GAPDH, and 40S ribosomal protein S11 (RPS11) utilized as the internal control [28,29]. Primers utilized in this study were as follows: myogenin forward (F) 5′-GCAAGGTGTGTAAGAGGAAG-3′, reverse (R) 5′-TGTGGGAGTTGCATTCACTG-3′; PRMT1 (PRMT1v1) F 5′-GCCTGCAAGTGAAGAGGAAC-3′, R 5′-CTCAGGACTGGTGGAGAAGC-3′; PRMT4 F 5′-ACCACACGGACTTCAAGGAC-3′, R 5′-CTCTTCACCAGGACCTCTGC-3′; PRMT5 F 5′- TCTCCCCACCAGCATTTTCC-3′, R 5′-TGGAGGGCGATTTTGGCTTA-3'; GAPDH F 5′- AACACTGAGCATCTCCCTCA-3′, R 5′-GTGGGTGCAGCGAACTTTAT-3′; 18S F 5′- GTAACCCGTTGAACCCCATT-3′, R 5′-CCATCCAATCGGTAGTAGCG-3′; RPS11 5′- CGTGACGAACATGAAGATGC-3′, R 5′-GCACATTGAATCGCACAGTC-3'.

### Mitochondrial respiration

At each timepoint of interest, C_2_C_12_ cells were washed with PBS and removed from the 100 mm × 20 mm culture plate using trypsin-EDTA. Two culture plates were combined together and the sample was centrifuged at 100×***g*** at room temperature for 3 min. The supernatant was discarded and the pellet was resuspended in mitochondrial respiration medium (MiR05) which contained: 0.5 mM EGTA, 10 mM KH_2_PO_4_, 3 mM MgCl_2_.6H_2_O, 60 mM potassium lactobionate, 20 mM HEPES, 20 mM taurine, 110 mM sucrose, and 1 g/l BSA. Cells were subsequently counted using Trypan Blue and a hemocytometer (Invitrogen). Samples were treated with 3 μg/10^6^ cells/ml digitonin for 5 min at 37°C on a digital rocker. Following permeabilization, samples were centrifuged at 800×***g*** for 3 min. The permeabilized cells were resuspended in MiR05 buffer and were used for determination of mitochondrial oxygen (O_2_) consumption at 37°C using the Oroboros Oxygraph-2 K (Oroboros Instruments Corp., Innsbruck, Austria). The cells (1 × 10^6^ cells/chamber) were placed into separate sealed chambers and the following substrates were added (volume; final concentration): glutamate (5 µl; 5 mM), followed by malate (4 µl; 2 mM), ADP (20 µl; 5 mM), cytochrome *c* (5 µl; 10 µM), pyruvate (5 µl; 5 mM), and succinate (20 µl; 20 mM). The rate of O_2_ consumption was recorded and expressed as picomoles/second/million cells using DatLab software (Oroboros Instruments Corp.).

### Statistical analyses

A one-way ANOVA with Bonferroni post-hoc test and Student’s *t*tests were used to identify differences between means during the myogenesis timecourse experiments. A two-way ANOVA with Bonferroni post-hoc tests was used for analysis of the PRMT1 inhibition experiments. Statistical analyses were performed on the raw datasets prior to the conversion to fold differences. Sample sizes for all experiments were *n*=3–6, with each *n* being the mean of two or three biological replicates. Statistical significance was accepted at *P*<0.05. Data in graphical summaries are presented as mean ± S.E.M.

## Results

### PRMT gene expression throughout myogenesis

**Figure 1:**
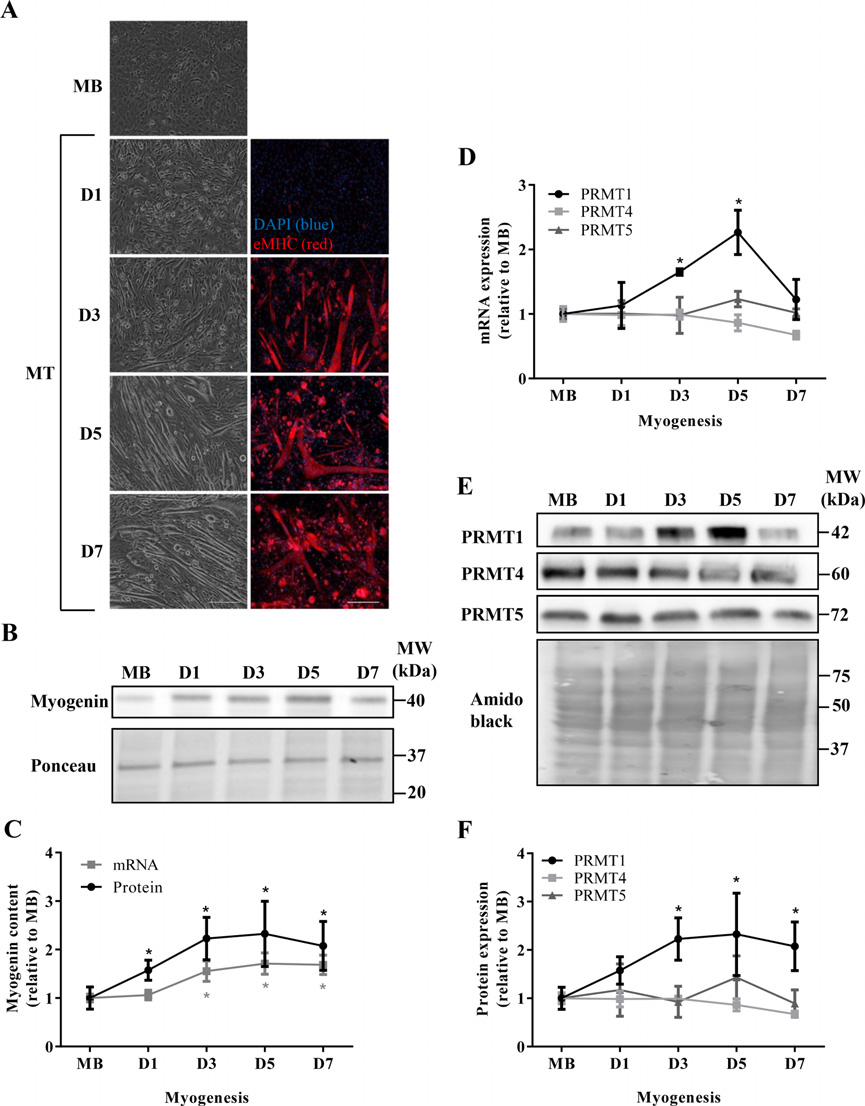
PRMT gene expression during myogenesis (**A**) Light microscope images (left column panels) of C_2_C_12_ MBs, and day 1 (D1), 3 (D3), 5 (D5), and 7 (D7) MT. Immunofluorescence images (right column panels) of muscle cells throughout the differentiation timecourse stained with DAPI (blue) and eMHC (red). Scale bar =200 μm. (**B**) Representative immunoblot (above) depicting myogenin levels throughout the experimental timecourse. Ponceau S stain (below) indicates equal loading between samples. (**C**) Graphical summary of *myogenin* mRNA (gray line) and protein (black line) expression levels across the timecourse of myogenesis. Data are displayed as relative to MB levels. *n*=5; *P<0.05 compared with MB mRNA content; *P<0.05 compared with MB protein content. (**D**) *PRMT1, PRMT4*, and *PRMT5* mRNA expression in MB, and D1, D3, D5, D7 MTs displayed relative MB levels. (**E**) Typical PRMT1, PRMT4, and PRMT5 immunoblots and amido black loading control image. (**F**) Graphical summary of PRMT protein content throughout C_2_C_12_ myogenesis. *n*=3–4; *P<0.05 compared with PRMT1 levels in MB.

To begin our characterization of PRMT expression and function during myogenesis, we first sought to confirm the progression of muscle development throughout the experimental timecourse. To this end, we examined cell morphology, eMHC expression, as well as myogenin transcript and protein levels, which all represent established histological and molecular markers of myogenesis [30,31]. Qualitative assessment of light microscopy images indicates that the C_2_C_12_ cells progressed morphologically from mononucleated MBs to robust MTs as expected throughout the differentiation timecourse (Figure 1A, left column panels). Immunofluorescence detection of eMHC also demonstrated the advancement of the myogenic program (Figure 1A, right column panels), as expression of the protein increased coincident with the fusion and growth of MTs throughout the 7-day timecourse. Furthermore, mRNA and protein levels of the myogenic regulatory factor myogenin increased as expected during the differentiation protocol (Figure 1B,C), which is indicative of typically progressing myogenesis. Myogenin mRNA content was ~1.5-fold higher after D3 relative to the MB stage (*P*<0.05). Myogenin protein content was significantly greater at all differentiating timepoints in comparison with the MB stage.

We next endeavored to examine the gene expression of PRMT1, PRMT4, and PRMT5 during the differentiation timecourse by measuring mRNA and protein levels of the enzymes. PRMT1 transcript levels were significantly higher at D5 compared with all preceding timepoints, and returned to baseline by D7 (Figure 1D). Both *PRMT4* and *PRMT5* mRNA levels remained unchanged throughout myogenesis (Figure 1D). The protein content of these enzymes followed their mRNA patterns of expression and were constitutively expressed across the timecourse (Figure 1E,F). In contrast, PRMT1 protein expression was significantly higher by D3 of differentiation, as compared with earlier timepoints, and remained elevated 2–2.2-fold until D7 (Figure 1E,F).

### PRMT activity during skeletal muscle development

**Figure 2:**
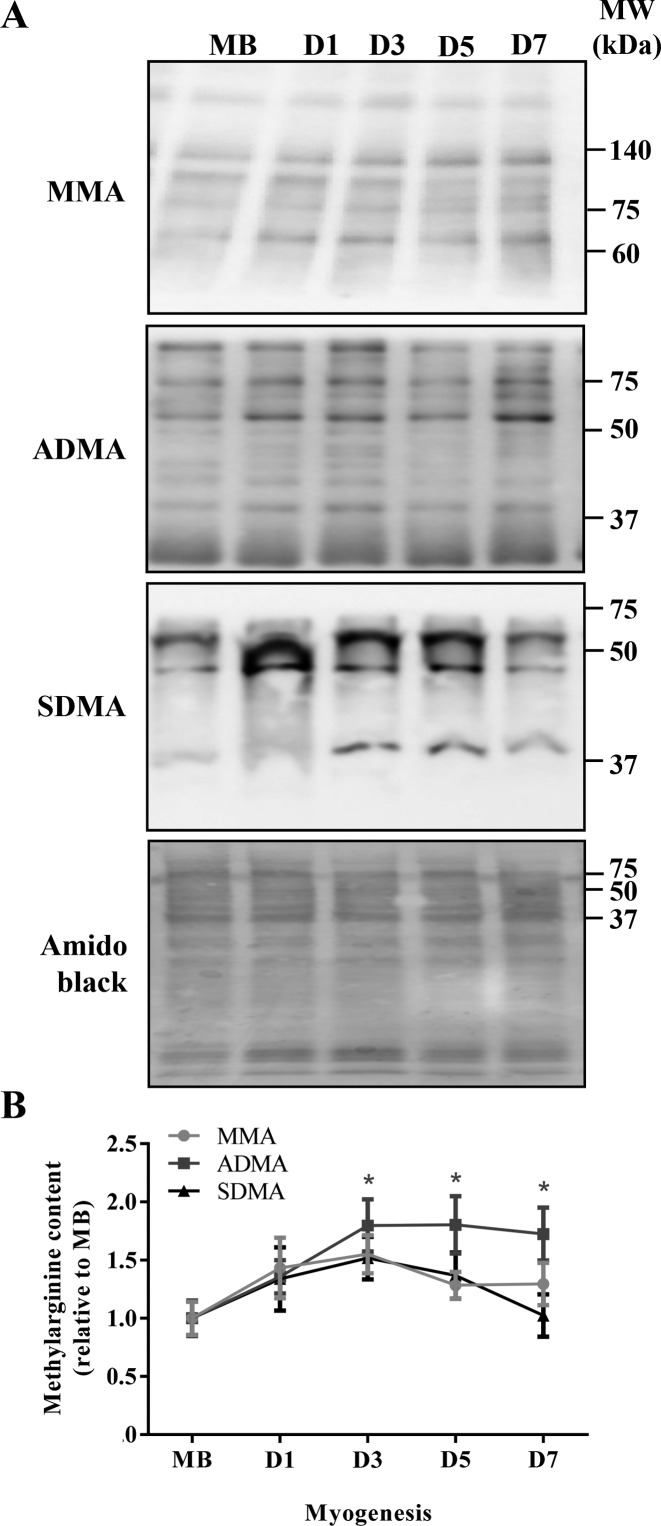
Global PRMT activity throughout myogenesis Representative (**A**) MMA, ADMA, and SDMA immunoblots at MB, and D1, D3, D5, and D7 stages of differentiation. A typical amido black stain is also shown to indicate consistent loading between samples. (**B**) Graphical summary of MMA, ADMA, and SDMA species, expressed relative to MB levels. *n*=5–7; *P<0.05 compared with MB ADMA content.

To determine the global activity of PRMTs during myogenesis, we utilized immunoblotting to probe for MMA, ADMA, and SDMA levels, which are established markers of PRMT activity, type I PRMT activity, and type II PRMT activity, respectively [1,32]. The three antibodies utilized in this experiment recognize unique methylarginine species, however within each species the immunoreagents do not identify specific methylated protein targets. For this analysis, therefore, we quantitated all the bands in a single lane for each sample and presented these data as an indicator of general methylarginine levels for each species. We hypothesized that PRMT activity assessed in this manner would reflect any myogenesis-induced alterations in PRMT protein content. ADMA content was increased by ~1.7-fold (*P*<0.05) at D3, as compared with the MB stage, and remained elevated (Figure 2A,B). In contrast, cellular MMA and SDMA levels did not change throughout myogenesis (Figure 2A,B).

**Figure 3:**
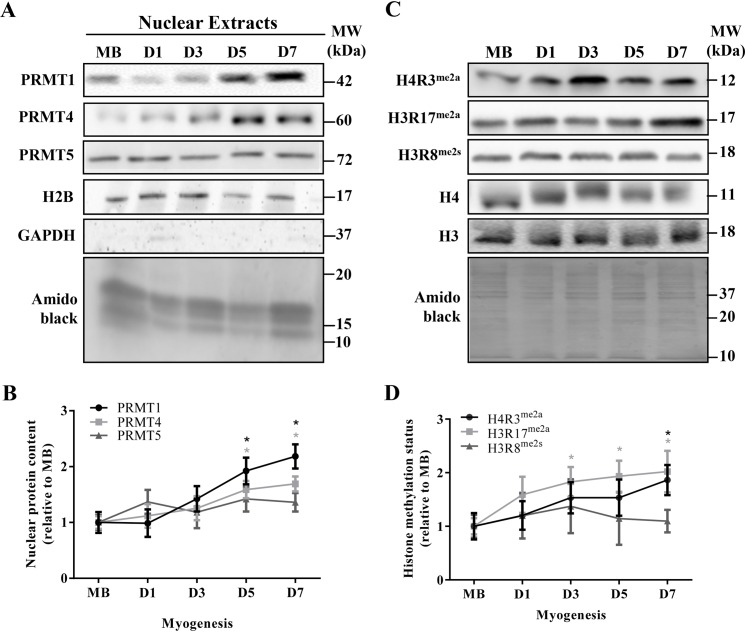
Specific PRMT methyltransferase activity during skeletal muscle differentiation (**A**) Representative PRMT1, PRMT4, PRMT5, H2B, and GAPDH immunoblots, as well as an amido black image of myonuclear lysates isolated from MB, D1, D3, D5, and D7 C_2_C_12_ cells. (**B**) Graphical depiction of myonuclear PRMT protein levels throughout myogenesis, expressed relative to the MB stage. *P<0.05 compared with MB PRMT1 content; *P<0.05 compared with MB PRMT4 content. (**C**) Typical immunoblots of H4R3^me2a^, H3R17^me2a^, H3R8^me2s^, total H4 and H3 protein content from MB, as well as D1, D3, D5, and D7 MTs. A representative amido black stain is presented below. (**D**) Graphical summary of histone arginine methylation status throughout myogenesis, depicted as the methylated form of the histone relative to the total histone amount, expressed relative to MB levels. *P<0.05 compared with MB H4R3^me2a^ status; *P<0.05 compared with MB H3R17^me2a^ status. *n*=4–7.

To examine PRMT-specific methyltransferase activities, we measured the methylated arginine levels of their histone targets. Asymmetric arginine dimethylation of H4R3^me2a^ and H3R17^me2a^, as well as symmetric arginine dimethylation of H3R8^me2s^, are specific and exclusive methylation targets of PRMT1, PRMT4, and PRMT5, respectively [33,34]. The myonuclear subfractions employed in this analysis were isolated with a high level of purity, as indicated by the presence of the nuclear protein H2B, and the absence of the cytosolic molecule GAPDH (Figure 3A). We first assessed myonuclear PRMT content during myogenesis since specific methyltransferase functions are dependent, in part, on the subcellular localization of the enzymes. Myonuclear PRMT1 and -4 accumulated throughout differentiation, demonstrating levels that were ~1.5–2.1-fold higher (*P*<0.05) at D5 and D7 compared with MB (Figure 3A,B). Nuclear PRMT5 content remained unchanged. Total H4 and H3 levels did not change over the course of muscle development (Figure 3C). Histone methylation status, expressed as the methylated form of the histone relative to the total amount of the protein, significantly increased during myogenesis for H4R3^me2a^ and H3R17^me2a^, reaching levels that were ~1.9-fold greater at D7 compared with MB (Figure 3C,D). In contrast, H3R8^me2s^ methylation status remained unchanged across myogenic development.

### PRMT1 function in myogenesis

**Figure 4:**
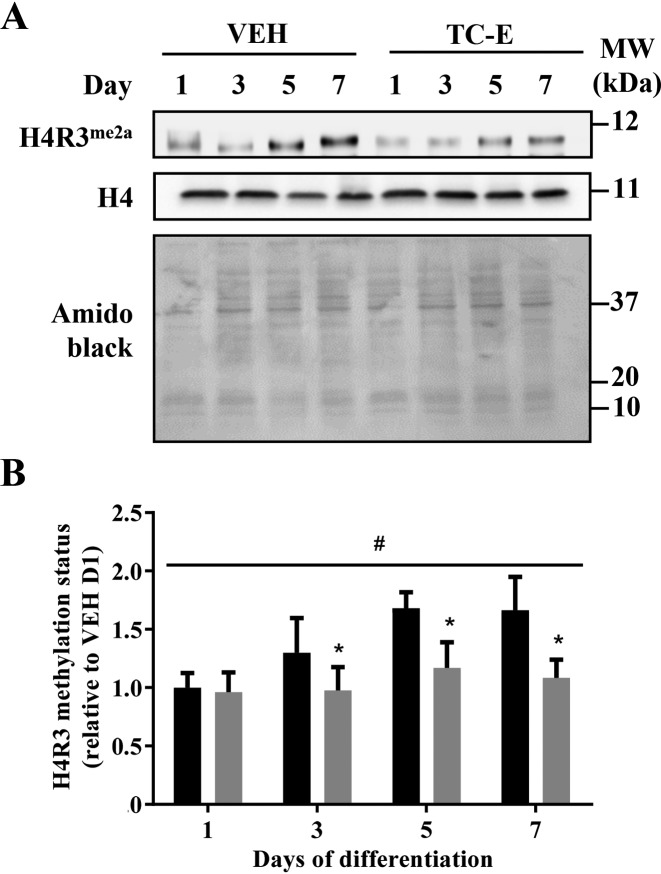
PRMT1 inhibition during myogenesis (**A**) Representative H4R3^me2a^ and H4 immunoblots, as well as amido black stain at C_2_C_12_ differentiation days 1, 3, 5, and 7 in the VEH and TC-E 5003 (TC-E) treatment conditions. (**B**) Graphical summary of H4R3 methylation status in the VEH and TC-E conditions expressed relative to levels in VEH D1. *n*=5; *P<0.05 compared with VEH at the same timepoint; ^#^*P*<0.05 main effect of time in the VEH.

In an effort to elucidate the role of PRMT1 in myogenesis, we employed the specific PRMT1 antagonist TC-E [23] to inhibit PRMT1 methyltransferase function during muscle differentiation. TC-E treatment did not affect cellular H4 content, which remained unchanged throughout myogenesis (Figure 4A). TC-E completely blocked the increase in H4R3^me2a^ methylation status that occurred with myogenic development (Figure 4A,B). H4R3^me2a^ methylation levels were significantly blunted by ~25% at D3–D7, thereby confirming the efficacy of the inhibitor.

**Figure 5:**
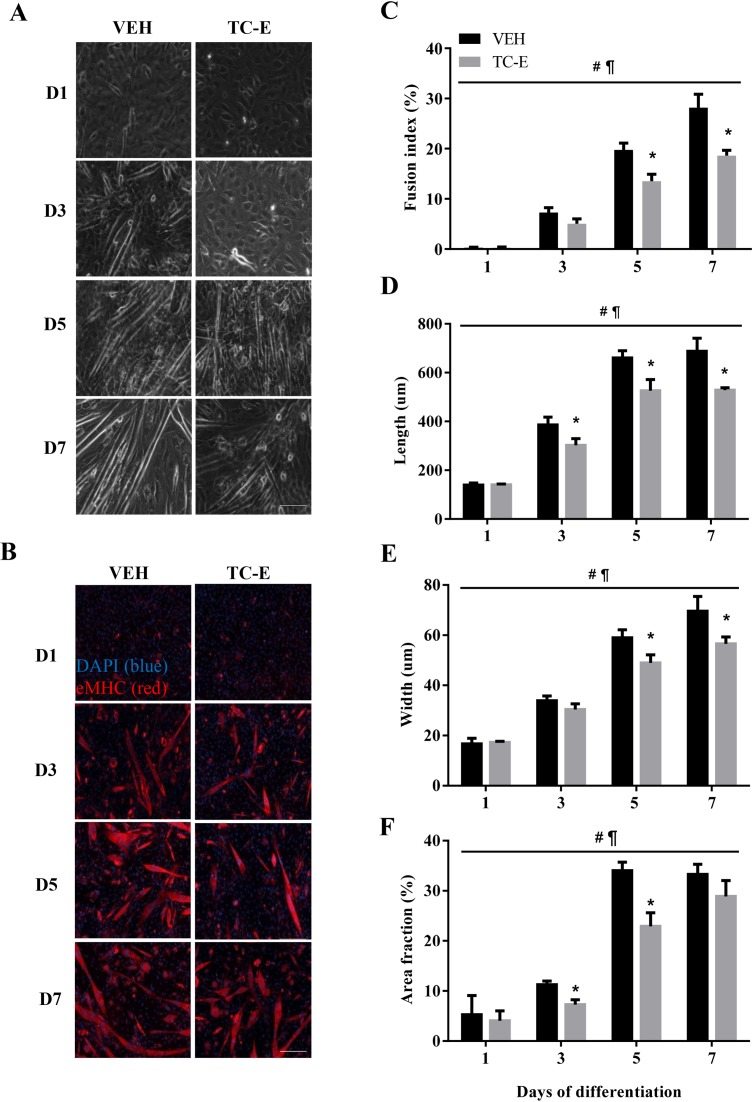
Effect of PRMT1 inhibition on the progression of myogenic differentiation (**A**) Light microscopy images of C_2_C_12_ muscle cells at D1, D3, D5, and D7 of differentiation in the VEH (left column) or TC-E condition (right column). (**B**) Immunofluorescence images stained with eMHC (red) and DAPI (blue) of VEH- (left column) and TC-E-treated cells (right column). Scale bar =200 μm. Graphical summaries of (**C**) MT fusion index, (**D**) MT length, (**E**) MT width, and (**F**) MT surface area of VEH- and TC-E-treated cells across the experimental timecourse. *n*=3–5; ^#^*P*<0.05 main effect of time in the VEH and TC-E conditions; ^¶^*P*<0.05 main effect of treatment; *P<0.05 compared with VEH at the same timepoint.

Light microscopy images revealed smaller and less robust MTs in the TC-E treated condition as compared with the VEH-treated cells (Figure 5A). To further provide evidence of the role of PRMT1 on muscle development, cellular morphology was quantitatively examined between the VEH and TC-E-treated conditions using eMHC and DAPI fluorescence analyses. As expected, there was a progressive increase in MT fusion, length, width, and surface coverage (i.e. area fraction) across the experimental timecourse in the VEH-treated cells (Figure 5B–F). This was also observed in the TC-E condition. However, TC-E treatment resulted in significant ~20–40% attenuations in all morphology metrics, which were observed at timepoints ranging from D3 to D7 of differentiation.

### Effects of PRMT1 inhibition on mitochondrial biogenesis and function

**Figure 6:**
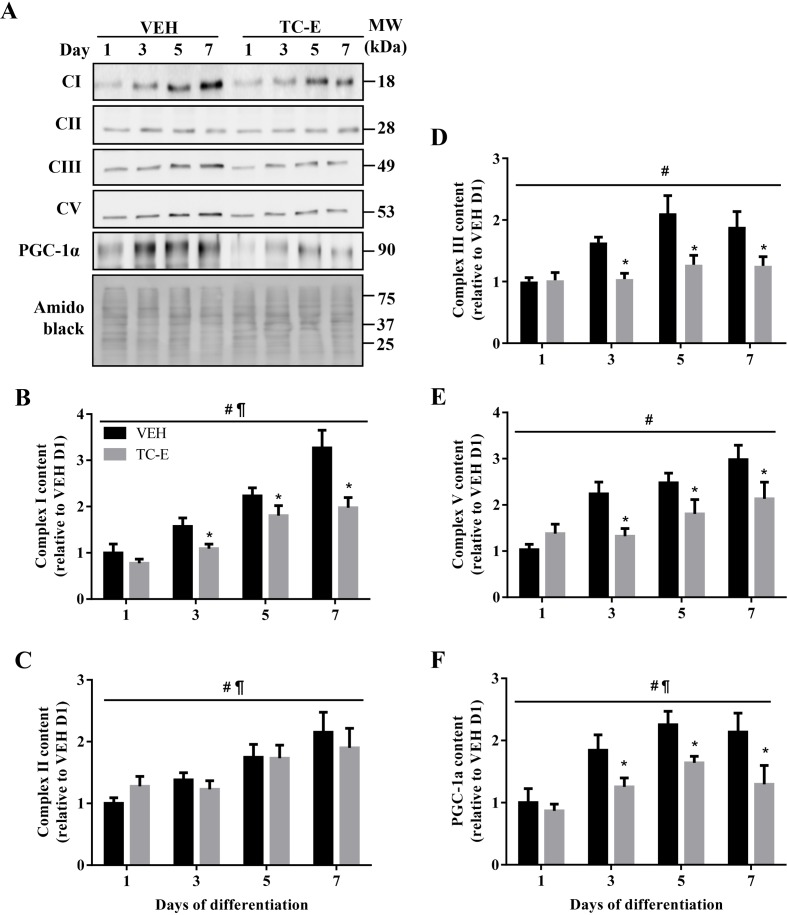
Effect of PRMT1 inhibition on mitochondrial biogenesis during muscle development (**A**) Representative immunoblots of mitochondrial complex I (CI), CII, CIII, CV, PGC-1α, as well as amido black stain, in VEH- and TC-E-treated cells across the differentiation timecourse. Graphical summaries of (**B**) CI, (**C**) CII, (**D**) CIII, (**E**) CV, and (**F**) PGC-1α protein content expressed relative to the levels in VEH D1 cells. *n*=4; ^#^*P*<0.05 main effect of time in the VEH and TC-E conditions; ^¶^*P*<0.05 main effect of treatment; *P<0.05 compared with VEH at the same timepoint.

To understand the potential mechanisms underlying the differentiation defects observed in PRMT1-inhibited cells, we examined the effects of TC-E on mitochondrial biogenesis and oxygen consumption. Indeed, mitochondrial content and function are required for optimal myogenic progression [35,36]. Protein levels of representative subunits of mitochondrial electron transport chain complex I (CI), CIII, and CV were attenuated by ~20–40% (*P*<0.05) in D3–D7 cells in response to PRMT1 inhibition (Figure 6A,B,D,E). In contrast, CII expression was similar between VEH and TC-E treatment conditions throughout myogenesis (Figure 6A,C). PRMT1 inhibition also resulted in the significant reduction by 25–40% in the protein content of PGC-1α, a master regulator of muscle mitochondrial biogenesis [37], during D3–D7 of differentiation (Figure 6A,F).

**Figure 7:**
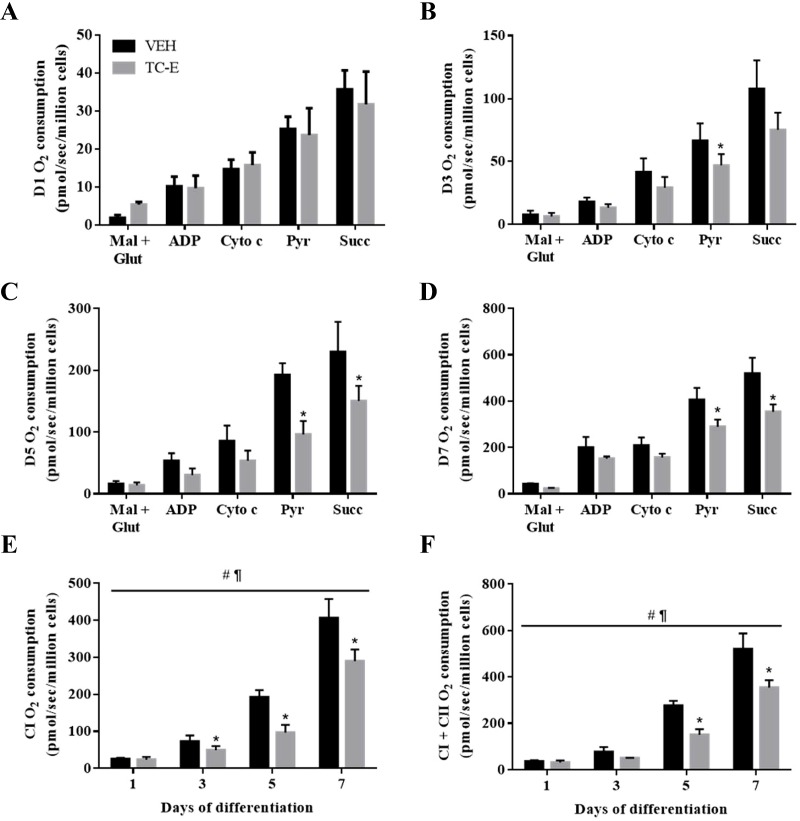
Effect of PRMT1 inhibition on mitochondrial respiration Graphical summaries of rates of mitochondrial oxygen consumption in (**A**) D1, (**B**) D3, (**C**) D5, and (**D**) D7 cells with the substrates malate and glutamate (Mal + Glut), ADP, cytochrome *c* (Cyto *c*), pyruvate (Pyr), and succinate (Succ) in VEH and TC-E treatment conditions. (**E**) CI and (**F**) CI + CII oxygen consumption values across the differentiation timecourse in the two experimental conditions. *n*=3–5; ^#^*P*<0.05 main effect of time in the VEH and TC-E conditions; ^¶^*P*<0.05 main effect of treatment; *P<0.05 compared with VEH-treated cells.

Finally, to further investigate the role of PRMT1 in mitochondrial biology, organelle respiration was measured during the timecourse of muscle development in the presence or absence of the PRMT1-specific antagonist. TC-E treatment did not affect CI- or CI + CII-driven mitochondrial oxygen consumption in D1 differentiated cells (Figure 7A,E,F). However, there was a ~20–35% reduction (*P*<0.05) in oxygen consumption in D3–D7 TC-E-treated cells following the addition of the CI substrate pyruvate, as compared with VEH-treated cells (Figure 7B–E). PRMT1 inhibition also significantly attenuated succinate/CI + CII supported mitochondrial respiration at D5 and D7 of myogenesis (Figure 7D,F).

## Discussion

The purpose of the current study was to provide a more comprehensive characterization of PRMT biology throughout the process of skeletal muscle differentiation. Our data revealed PRMT-specific patterns of expression and activity during myogenesis, which suggest individualized contributions for each enzyme to the muscle development process. Moreover, complementary assessments of PRMT1, -4, and -5 function indicate that PRMT methyltransferase activity in muscle is substrate specific, depending in part, on the subcellular location of the protein target. Since PRMT1 expression and activity were particularly responsive to myogenic cues, we inhibited its methyltransferase activity in order to elucidate its role in skeletal muscle differentiation. Muscle cells in which PRMT1 activity was knocked down exhibited differentiation defects that were associated with attenuated mitochondrial biogenesis and respiratory function. Thus, extending recent work implicating the requirement of PRMT1 in skeletal muscle regeneration *in vivo* [17], our results demonstrate that PRMT1 is necessary during differentiation in order to evoke complete myogenic development, however other PRMTs including PRMT4 and -5 are likely also involved in this process. Moreover, the negative effects of PRMT1 inhibition on myogenesis are at least partially mitochondrially mediated. The present study enhances our understanding of PRMT1, -4, and -5 biology during the plasticity of skeletal muscle development, as well as provides additional mechanistic evidence for a role of PRMT1 in driving the myogenic program.

Previous studies have demonstrated that several members of the PRMT family are expressed during skeletal muscle cell development, including PRMT1, -4, -5, and -7 [16,18,19,38]. Unfortunately however, a coherent understanding of PRMT transcript and protein levels during myogenesis has been difficult to achieve due, in part, to the disparate models and timing utilized thus far. Therefore, we sought to address this knowledge gap by clarifying PRMT gene expression via the employment of a standardized, comprehensive timecourse of C_2_C_12_ skeletal muscle cell differentiation. Our data revealed a measure of PRMT specificity with respect to gene expression during myogenesis. PRMT1 was induced at the mRNA and protein levels, while PRMT4 and -5 remained unchanged. PRMT1 followed a similar pattern of expression as the myogenic regulatory factor myogenin, which suggests a common upstream regulator. A candidate may be Eya1, which affects myogenin expression [39], and was recently demonstrated to be part of a myogenic pathway involving PRMT1 [17]. However, our data contradict this recent study by Blanc et al. [17], where the authors observed that *PRMT1* mRNA and protein content were similar between proliferating and differentiating isolated primary muscle stem cells. The discrepancy between their results and those of the current study may be attributed to differences in cell type and/or duration of the myogenic timecourse utilized. In line with our findings, previous studies reported that PRMT4 and -5 transcript and protein levels were constitutively expressed during early and later points of skeletal muscle development [4,18,19]. Constitutive expression of PRMT5 appears to be consistent with its roles in both the proliferative and differentiation phases of skeletal muscle development [15,40]. The differential expression pattern that we observed between PRMTs during myogenesis is likely due, in part, to unique, PRMT-specific upstream regulatory mechanisms.

We utilized two complementary approaches to investigate PRMT function during myogenesis. We first examined global PRMT activity by assessing total cellular MMA, ADMA, and SDMA levels. The MMA mark is a measure of non-specific PRMT activity, while the accumulation of ADMA and SDMA marks are indicative of type I and type II PRMT function, respectively [1,41]. Furthermore, since PRMT1 and PRMT5 catalyze the majority of ADMA and SDMA producing reactions, respectively [33,41,42], the appearance of these marks generally reflects the activities of these enzymes. A notable caveat is that relative PRMT activities have not yet been elucidated in skeletal muscle. We observed an increase in ADMA levels coincident with unchanged amounts of MMA and SDMA methylarginine species during the progression of muscle differentiation. The significant up-regulation in ADMA content was consistent with the rise in PRMT1 protein content. Previous evidence supports the idea that enzymatic activities of PRMTs are altered during muscle development [4,14,17]. It is interesting to note here that, similar to our PRMT4 results, others have also reported alterations in PRMT activity during myogenesis that are independent from any lack of change in PRMT content [4]. Certainly, activation of PRMTs may be facilitated by a number of mechanisms in this scenario, including the subcellular translocation of existing enzymes to the appropriate compartment, as well as stimulatory protein–protein interactions or post-translational modifications [14,18,43].

We isolated myonuclei and assessed the targetted methyltransferase activities of PRMT1, -4, and -5 throughout the experimental myogenic timecourse. The ADMA marks on H4R3^me2a^ and H3R17^me2a^, as well as the SDMA deposited on H3R8^me2s^, are specifically and exclusively catalyzed by PRMT, -4, and -5, respectively [10,33]. We found that the elevation in targetted PRMT activities generally reflected the increased nuclear accretion of the enzymes. Interestingly, while cellular PRMT4 content remained unchanged during differentiation, we observed a specific subcellular redistribution of PRMT4 within myonuclei, which corresponded with increased H3R17^me2a^ methylation status. These observations are in line with earlier work that examined the nuclear function of PRMT4 [18]. It must also be highlighted that each PRMT has many putative arginine methylation targets, and each member can have differential preferences for its targets [33]. For instance, the interaction between PRMT5 and COPR5 causes PRMT5 to alter its specificity to preferentially methylate H4R3^me2s^ over H3R8^me2s^ [44]. Altogether, our data suggest that PRMTs are active methyl donors throughout myogenesis and demonstrate specificity for their histone and non-histone targets in skeletal muscle.

Relative to other PRMTs, PRMT4 and -5 expression and function during myogenesis have been extensively studied [18–20,22,38,45]. Work from George Muscat’s laboratory was the first to demonstrate a role for PRMTs, specifically PRMT4, in potentiating myogenesis, clearly supporting a positive function of arginine methylation in mammalian differentiation [18]. By contrast, there are few reports that have directly assessed the necessity of PRMT1 throughout skeletal muscle development [4,17,43]. In an effort to expand our understanding of the myogenic functions of PRMT1, the enzyme that accounts for the majority (>80%) of arginine methyltransferase activity [46], we pharmacologically inhibited PRMT1 with TC-E and evaluated its impact on myogenesis. The strategy to employ a drug antagonist of PRMT1 was selected here in part because the use of small molecules is a more clinically translatable application, as compared with the use of RNA-based technologies (e.g. siRNA, shRNA) or transgenic approaches (e.g. overexpression or knockout animals). Moreover, TC-E possesses remarkably high specificity for PRMT1, as compared with other arginine and lysine methyltransferases [23]. The compound demonstrates potent anticancer activity and inhibition of androgen-dependent transcription in MCF7a and LNCaP cells, indicative of attenuated PRMT1 function. In skeletal muscle cells, TC-E treatment was able to completely block the differentiation-associated increase in PRMT1 activity, as evidenced by H4R3^me2a^ methylation status, indicating that the drug successfully inhibited PRMT1. Our data demonstrating that TC-E treatment significantly attenuated various morphological metrics of C_2_C_12_ differentiation reveals that PRMT1 activity is required for the optimal progression of the myogenic program. These results confirm recent work by Blanc et al. [17] who showed that PRMT1 is essential to successful muscle regeneration *in vivo* in response to cytotoxic injury. Our work also builds on, and extends these *in vivo* data by demonstrating that inhibition of PRMT1 exclusively during and after the onset of differentiation, as compared with initiating the knockout prior to the myogenic stimulus (i.e. cardiotoxin injury), reveals a role for the enzyme specifically during muscle differentiation.

Mitochondrial biogenesis is necessary for the myogenic program to proceed under both *in vitro* and *in vivo* conditions. For example, MBs that lack mtDNA fail to differentiate into MTs [47]. Moreover, skeletal muscle regeneration is characterized by a nearly five-fold increase in mitochondrial content during the onset of muscle differentiation [48]. We suspected that a potential mechanism linking PRMT1 inhibition to the observed differentiation defects was mitochondrially mediated. Our rationale was based on previous studies implicating PRMT1 [49,50] and arginine methyltransferase activity [51] in mitochondrial biogenesis and function. Indeed, Teyssier et al. [50] very elegantly demonstrated more than a decade ago that PRMT1 methylates PGC-1α, a master regulator of muscle plasticity and mitochondrial biogenesis, which directly stimulates the transcriptional function of the co-activator. More recent work from Sha et al. [49] showed that PRMT1 is almost entirely responsible for depositing the ADMA mark on mitochondrial proteins, and that PRMT1 knockdown resulted in reduced mitochondrial respiratory activity, ATP synthesis, as well as a significant elevation in oxidant production. Consistent with these reports, we observed that PRMT1 inhibition led to attenuated mitochondrial biogenesis and function in skeletal muscle cells. Although we were unable to resolve the OXPHOS complex IV subunit with a reliable degree of confidence, representative protein subunits from complexes I, III, and V were significantly lower in the TC-E-treated cells during days 3–7 of differentiation. Interestingly, the rise in complex II content during myogenesis was unaffected by PRMT1 inhibition. The OXPHOS protein content data were reflected by results from the mitochondrial oxygen consumption trials, which demonstrated attenuated organelle respiratory function when PRMT1 activity was blunted. It is likely that complex II was largely spared due, in part, to its composition being solely dependent on nuclear DNA-encoded subunits, as well as to its dedicated assembly apparatus [52].

PGC-1α levels were also blunted as a function of PRMT1 inhibition in skeletal muscle. The co-activator stimulates the transcription of mitochondrial genes located in nuclear and mitochondrial genomes by, for example interacting with nuclear respiratory factor 1 and mitochondrial transcription factor A (Tfam), respectively [53,54]. Notably, PGC-1α also participates in an autoregulatory positive feedback loop driving its own expression [55], as well as contributes to the transcriptional activation of Tfam [56]. It is therefore reasonable to posit that the attenuated PGC-1α expression in response to PRMT1 inhibition caused a dysregulation in Tfam expression and/or function. The expression of mtDNA genes encoding OXPHOS subunits is critical for maintaining proper function of the organelle, as evidenced by the considerable impairments caused by mtDNA mutations [54]. Thus, although speculative, our results suggest that PRMT1 inhibition likely affects mitochondrial content and function via decrements in PGC-1α content and/or activity, with particular consequence on events occurring within the organelle. Additional studies should also address whether the decrement in mitochondrial biogenesis pathways are a direct effect of PRMT1 inhibition or potentially secondary to the pharmacologically induced attenuation of muscle differentiation, or a combination thereof.

In summary, our results demonstrate that the expression and activities of PRMT1, -4, and -5 display differential responses during skeletal muscle development. Indeed, PRMT1 biology was particularly responsive to myogenic cues. We also show that inhibition of PRMT1 in skeletal muscle cells results in morphological deficiencies, as well as decrements in mitochondrial biogenesis and respiratory function. Thus, complementing recent *in vivo* work [17], this investigation supports a critical role for PRMT1 specifically in the optimal progression of muscle differentiation. The present study enhances our understanding of PRMT biology during skeletal muscle plasticity elicited by myogenesis and identifies a mitochondrially mediated mechanism that links PRMT1 inhibition to defects in skeletal muscle development.

## Abbreviations

ADMA: asymmetric dimethylarginine
CI, II, III, IV, V: mitochondrial complex I, II, II, IV, and IV
COPR5: cooperator of PRMT5
DM: differentiation medium
DMEM: Dulbecco’s modified Eagle’s medium
eMHC: embryonic myosin heavy chain
GM: growth medium
H2B: histone 2B
H3R8^me2s^: histone 3 arginine 8 symmetric dimethylation
H3R17^me2a^: histone 3 arginine 17 asymmetric dimethylation
H4R3^me2a^: histone 4 arginine 3 asymmetric dimethylation
MB: myoblast
MiR05: mitochondrial respiration medium
MMA: monomethylarginine
MT: myotube
NET: nuclear extraction buffer
OXPHOS: oxidative phosphorylation
PGC-1α: peroxisome proliferator-activated receptor γ co-activator-1α
PRMT: protein arginine methyltransferase
qPCR: quantitative polymerase chain reaction
RIPA: radioimmunoprecipitation assay
RPS11: 40S ribosomal protein S11
SDMA: symmetric dimethylarginine
STM: cytoplasmic extraction buffer
TBST: TBS-Tween 20
TC-E: TC-E 5003 ; N,N'-(Sulfonyldi-4,1-phenylene)bis(2-chloroacetamide)
Tfam: mitochondrial transcription factor A
VEH: vehicle

## References

[1] Bedford M.T. and Clarke S.G. (2009) Protein arginine methylation in mammals: who, what, and why. Mol. Cell. 33, 1–13 10.1016/j.molcel.2008.12.013 19150423 PMC3372459

[2] Cha B. and Jho E.-H. (2012) Protein arginine methyltransferases (PRMTs) as therapeutic targets. Expert Opin. Ther. Targets 16, 651–664 10.1517/14728222.2012.688030 22621686

[3] Stouth D.W., van Lieshout T.L., Shen N.Y. and Ljubicic V. (2017) Regulation of skeletal muscle plasticity by protein arginine methyltransferases and their potential roles in neuromuscular disorders. Front. Physiol. 8, 870 10.3389/fphys.2017.00870 29163212 PMC5674940

[4] Kim S.J., Yoo B.C., Uhm C.S. and Lee S.W. (2011) Posttranslational arginine methylation of lamin A/C during myoblast fusion. Biochim. Biophys. Acta 1814, 308–317 10.1016/j.bbapap.2010.11.006 21111849

[5] Wei H., Mundade R., Lange K. and Lu T. (2014) Protein arginine methylation of non-histone proteins and its role in diseases. Cell Cycle 13, 32–41 10.4161/cc.27353 24296620 PMC3925732

[6] Larsen S.C., Sylvestersen K.B., Mund A., Lyon D., Mullari M., Madsen M.V. (2016) Proteome-wide analysis of arginine monomethylation reveals widespread occurrence in human cells. Sci. Signal. 9, http://stke.sciencemag.org/content/9/443/rs9.long 10.1126/scisignal.aaf7329 27577262

[7] Pawlak M.R., Scherer C.A., Chen J., Roshon M.J. and Ruley H.E. (2000) Arginine N-methyltransferase 1 is required for early postimplantation mouse development, but cells deficient in the enzyme are viable. Mol. Cell. Biol. 20, 4859–4869 10.1128/MCB.20.13.4859-4869.2000 10848611 PMC85937

[8] Yadav N., Lee J., Kim J., Shen J., Hu M.C.-T., Aldaz C.M. (2003) Specific protein methylation defects and gene expression perturbations in coactivator-associated arginine methyltransferase 1-deficient mice. Proc. Natl. Acad. Sci. U.S.A. 100, 6464–6468 10.1073/pnas.1232272100 12756295 PMC164469

[9] Tee W.-W., Pardo M., Theunissen T.W., Yu L., Choudhary J.S., Hajkova P. (2010) Prmt5 is essential for early mouse development and acts in the cytoplasm to maintain ES cell pluripotency. Genes Dev. 24, 2772–2777 10.1101/gad.606110 21159818 PMC3003195

[10] Yang Y. and Bedford M.T. (2012) Protein arginine methyltransferases and cancer. Nat. Rev. Cancer 10.1038/nrc3409 23235912

[11] Reporter M. and Corbin J.L. (1971) NG, NG-Dimethylarginine in myosin during muscle development. Biochem. Biophys. Res. Commun. 43, 644–650 10.1016/0006-291X(71)90663-2 5563312

[12] Stouth D.W., Manta A. and Ljubicic V. (2017) Protein arginine methyltransferase expression, localization, and activity during disuse-induced skeletal muscle plasticity. Am. J. Physiol. Cell Physiol. 10.1152/ajpcell.00174.2017 29092819 PMC5866438

[13] vanLieshout T.L., Stouth D.W., Tajik T. and Ljubicic V. (2017) Exercise-induced protein arginine methyltransferase expression in skeletal muscle. Med. Sci. Sports Exerc. 10.1249/MSS.0000000000001476 29112628

[14] Kawabe Y.I., Wang Y.X., McKinnell I.W., Bedford M.T. and Rudnicki M.A. (2012) Carm1 regulates Pax7 transcriptional activity through MLL1/2 recruitment during asymmetric satellite stem cell divisions. Cell Stem Cell 11, 333–345 10.1016/j.stem.2012.07.001 22863532 PMC3438319

[15] Zhang T., Günther S., Looso M., Künne C., Krüger M., Kim J. (2015) Prmt5 is a regulator of muscle stem cell expansion in adult mice. Nat. Commun. 6, 7140 10.1038/ncomms8140 26028225 PMC4458870

[16] Blanc R.S., Vogel G., Chen T., Crist C. and Richard S. (2016) PRMT7 preserves satellite cell regenerative capacity. Cell Rep. 14, 1528–1539 10.1016/j.celrep.2016.01.022 26854227

[17] Blanc R.S., Vogel G., Li X., Yu Z., Li S. and Richard S. (2017) Arginine methylation by PRMT1 regulates muscle stem cell fate. Mol. Cell. Biol. 37, e00457–16 10.1128/MCB.00457-16 27849571 PMC5247616

[18] Chen S.L., Loffler K.A., Chen D., Stallcup M.R. and Muscat G.E.O. (2002) The coactivator-associated arginine methyltransferase is necessary for muscle differentiation: CARM1 coactivates myocyte enhancer factor-2. J. Biol. Chem. 277, 4324–4333 10.1074/jbc.M109835200 11713257

[19] Wang S.M., Dowhan D.H., Eriksson N.A. and Muscat G.E.O. (2012) CARM1/PRMT4 is necessary for the glycogen gene expression programme in skeletal muscle cells. Biochem. J. 444, 323–331 10.1042/BJ20112033 22428544

[20] Dacwag C.S., Bedford M.T., Sif S. and Imbalzano A.N. (2009) Distinct protein arginine methyltransferases promote ATP-dependent chromatin remodeling function at different stages of skeletal muscle differentiation. Mol. Cell. Biol. 29, 1909–1921 10.1128/MCB.00742-08 19188441 PMC2655603

[21] Batut J., Duboé C. and Vandel L. (2011) The methyltransferases PRMT4/CARM1 and PRMT5 control differentially myogenesis in zebrafish. PLoS ONE 6, e25427 22016767 10.1371/journal.pone.0025427 PMC3189919

[22] Dacwag C.S., Ohkawa Y., Pal S., Sif S. and Imbalzano A.N. (2007) The protein arginine methyltransferase Prmt5 is required for myogenesis because it facilitates ATP-dependent chromatin remodeling. Mol. Cell. Biol. 27, 384–394 10.1128/MCB.01528-06 17043109 PMC1800640

[23] Bissinger E.-M., Heinke R., Spannhoff A., Eberlin A., Metzger E., Cura V. (2011) Acyl derivatives of p-aminosulfonamides and dapsone as new inhibitors of the arginine methyltransferase hPRMT1. Bioorg. Med. Chem. 19, 3717–3731 10.1016/j.bmc.2011.02.032 21440447

[24] Dimauro I., Pearson T., Caporossi D. and Jackson M.J. (2012) A simple protocol for the subcellular fractionation of skeletal muscle cells and tissue. BMC Res. Notes 5, 513 10.1186/1756-0500-5-513 22994964 PMC3508861

[25] Romero-Calvo I., Ocón B., Martínez-Moya P., Suárez M.D., Zarzuelo A., Martínez-Augustin O. (2010) Reversible Ponceau staining as a loading control alternative to actin in Western blots. Anal. Biochem. 401, 318–320 10.1016/j.ab.2010.02.036 20206115

[26] Ghosh R., Gilda J.E. and Gomes A.V. (2014) The necessity of and strategies for improving confidence in the accuracy of western blots. Expert Rev. Proteomics 11, 549–560 10.1586/14789450.2014.939635 25059473 PMC4791038

[27] Fortes M.A.S., Marzuca-Nassr G.N., Vitzel K.F., Da Justa Pinheiro C.H., Newsholme P., Curi R. (2016) How useful are they in skeletal muscle diabetes studies and muscle hypertrophy models? Anal. Biochem. 504, 38–40 10.1016/j.ab.2016.03.023 27060530

[28] Manzano R., Toivonen J.M., Calvo A.C., Miana-Mena F.J., Zaragoza P., Muñoz M.J. (2011) Sex, fiber-type, and age dependent in vitro proliferation of mouse muscle satellite cells. J. Cell. Biochem. 112, 2825–2836 10.1002/jcb.23197 21608019

[29] Hildyard J.C.W. and Wells D.J. (2014) Identification and validation of quantitative PCR reference genes suitable for normalizing expression in normal and dystrophic cell culture models of myogenesis. PLoS Curr. 6, 10.1371/currents.md.faafdde4bea8df4aa7d06cd5553119a6 PMC3948689 24634799

[30] Hawke T.J. and Garry D.J. (2001) Myogenic satellite cells: physiology to molecular biology. J. Appl. Physiol. 91, http://jap.physiology.org/content/91/2/534.long 11457764 10.1152/jappl.2001.91.2.534

[31] Yin H., Price F. and Rudnicki M. (2013) Satellite cells and the muscle stem cell niche. Physiol. Rev. 93, 23–67 10.1152/physrev.00043.2011 23303905 PMC4073943

[32] Bedford M.T. and Richard S. (2005) Arginine methylation: an emerging regulator of protein function. Mol. Cell 18, 263–272 10.1016/j.molcel.2005.04.003 15866169

[33] Di Lorenzo A. and Bedford M.T. (2011) Histone arginine methylation. FEBS Lett. 585, 2024–2031 10.1016/j.febslet.2010.11.010 21074527 PMC3409563

[34] Blanc R.S. and Richard S. (2017) Arginine methylation: the coming of age. Mol. Cell 65, 8–24 10.1016/j.molcel.2016.11.003 28061334

[35] Larsson N.-G., Wang J., Wilhelmsson H., Oldfors A., Rustin P., Lewandoski M. (1998) Mitochondrial transcription factor A is necessary for mtDNA maintance and embryogenesis in mice. Nat. Genet. 18, 231–236 10.1038/ng0398-231 9500544

[36] Collu-Marchese M., Shuen M., Pauly M., Saleem A. and Hood D.A. (2015) The regulation of mitochondrial transcription factor A (Tfam) expression during skeletal muscle cell differentiation. Biosci. Rep. 35 10.1042/BSR20150073 PMC4613705 26182383

[37] Jornayvaz F.R. and Shulman G.I. (2010) Regulation of mitochondrial biogenesis. Essays Biochem. 47, 69–84 10.1042/bse0470069 20533901 PMC3883043

[38] Paul C., Sardet C. and Fabbrizio E. (2012) The histone- and PRMT5-associated protein COPR5 is required for myogenic differentiation. Cell Death Differ. 19, 900–908 10.1038/cdd.2011.193 22193545 PMC3321631

[39] Le Grand F., Grifone R., Mourikis P., Houbron C., Gigaud C., Pujol J. (2012) Six1 regulates stem cell repair potential and self-renewal during skeletal muscle regeneration. J. Cell Biol. 198, http://jcb.rupress.org/content/198/5/815?ijkey=197a484fa738a98000c477a3a5451d1e0288ee9f&keytype2=tf_ipsecsha 10.1083/jcb.201201050 22945933 PMC3432771

[40] Mallappa C., Hu Y.J., Shamulailatpam P., Tae S., Sif S. and Imbalzano A.N. (2011) The expression of myogenic microRNAs indirectly requires protein arginine methyltransferase (Prmt)5 but directly requires Prmt4. Nucleic Acids Res. 39, 1243–1255 10.1093/nar/gkq896 20947566 PMC3045594

[41] Dhar S., Vemulapalli V., Patananan A.N., Huang G.L., Di Lorenzo A., Richard S. (2013) Loss of the major type I arginine methyltransferase PRMT1 causes substrate scavenging by other PRMTs, Sci. Rep. 3, 1311 23419748 10.1038/srep01311 PMC3575585

[42] Tang J., Frankel A., Cook R.J., Kim S., Paik W.K., Williams K.R. (2000) PRMT1 is the predominant type I protein arginine methyltransferase in mammalian cells. J. Biol. Chem. 275, 7723–7730 10.1074/jbc.275.11.7723 10713084

[43] Iwasaki H. and Yada T. (2007) Protein arginine methylation regulates insulin signaling in L6 skeletal muscle cells. Biochem. Biophys. Res. Commun. 364, 1015–1021 10.1016/j.bbrc.2007.10.113 17971302

[44] Lacroix M., El Messaoudi S., Rodier G., Le Cam A., Sardet C. and Fabbrizio E. (2008) The histone-binding protein COPR5 is required for nuclear functions of the protein arginine methyltransferase PRMT5. EMBO Rep. 9, 452–458 10.1038/embor.2008.45 18404153 PMC2373370

[45] Bentzinger C.F., Wang Y.X. and Rudnicki M.A. (2012) Building muscle: molecular regulation of myogenesis. Cold Spring Harb. Perspect. Biol. 4, a008342 10.1101/cshperspect.a008342 22300977 PMC3281568

[46] Wei H., Mundade R., Lange K.C. and Lu T. (2014) Protein arginine methylation of non-histone proteins and its role in diseases. Cell Cycle 13, 32–41 10.4161/cc.27353 24296620 PMC3925732

[47] Herzberg N.H., Zwart R., Wolterman R.A., Ruiter J.P.N., Wanders R.J.A., Bolhuis P.A. (1993) Differentiation and proliferation of respiration-deficient human myoblasts. Biochim. Biophys. Acta 1181, 63–67 10.1016/0925-4439(93)90091-E 7681329

[48] Duguez S., Féasson L., Denis C. and Freyssenet D. (2002) Mitochondrial biogenesis during skeletal muscle regeneration. Am. J. Physiol. Endocrinol. Metab. 282, http://ajpendo.physiology.org/content/282/4/E802.long 10.1152/ajpendo.00343.2001 11882500

[49] Sha L., Daitoku H., Araoi S., Kaneko Y., Takahashi Y., Kako K. (2017) Asymmetric arginine dimethylation modulates mitochondrial energy metabolism and homeostasis in *Caenorhabditis elegans*. Mol. Cell. Biol. 10.1128/MCB.00504-16 27994012 PMC5335503

[50] Teyssier C., Ma H., Emter R., Kralli A. and Stallcup M.R. (2005) Activation of nuclear receptor coactivator PGC-1alpha by arginine methylation. Genes Dev. 19, 1466–1473 10.1101/gad.1295005 15964996 PMC1151663

[51] Rhein V.F., Carroll J., Ding S., Fearnley I.M. and Walker J.E. (2013) NDUFAF7 methylates Arginine 85 in the NDUFS2 subunit of human complex I, J. Biol. Chem., 288, 33016–33026 24089531 10.1074/jbc.M113.518803 PMC3829151

[52] Rutter J., Winge D.R. and Schiffman J.D. (2010) Succinate dehydrogenase - assembly, regulation and role in human disease. Mitochondrion 10, 393–401 10.1016/j.mito.2010.03.001 20226277 PMC2874626

[53] Hood D.A., Tryon L.D., Carter H.N., Kim Y. and Chen C.C.W. (2016) Unravelling the mechanisms regulating muscle mitochondrial biogenesis. Biochem. J. 473, 10.1042/BCJ20160009 27470593

[54] Scarpulla R.C. (2008) Transcriptional paradigms in mammalian mitochondrial biogenesis and function. Physiol. Rev. 88, 10.1152/physrev.00025.2007 18391175

[55] Handschin C., Rhee J., Lin J., Tarr P.T. and Spiegelman B.M. (2003) An autoregulatory loop controls peroxisome proliferator-activated receptor gamma coactivator 1alpha expression in muscle. Proc. Natl. Acad. Sci. U.S.A. 100, 7111–7116 10.1073/pnas.1232352100 12764228 PMC165838

[56] Wu Z., Puigserver P., Andersson U., Zhang C., Adelmant G., Mootha V. (1999) Mechanisms controlling mitochondrial biogenesis and respiration through the thermogenic coactivator PGC-1. Cell 98, 115–124 10.1016/S0092-8674(00)80611-X 10412986

